# Hydrothermal Synthesis and In Vivo Fluorescent Bioimaging Application of Eu^3+^/Gd^3+^ Co-Doped Fluoroapatite Nanocrystals

**DOI:** 10.3390/jfb13030108

**Published:** 2022-07-29

**Authors:** Sriyani Menike Korale Gedara, Zi-You Ding, Iresha Lakmali Balasooriya, Yingchao Han, Merita Nirmali Wickramaratne

**Affiliations:** 1 State Key Laboratory of Advanced Technology for Material Synthesis and Processing, Wuhan University of Technology, Wuhan 430070, China; sriyanimenike@gmail.com (S.M.K.G.); dingziyou@hotmail.com (Z.-Y.D.); iresha.balasooriya@gmail.com (I.L.B.); 2 Foshan Xianhu Laboratory of the Advanced Energy Science and Technology Guangdong Laboratory, Xianhu Hydrogen Valley, Foshan 528200, China; 3 Faculty of Medicine, Sabaragamuwa University of Sri Lanka, Belihuloya 70140, Sri Lanka; meritanirmali@gmail.com

**Keywords:** fluoroapatite, rare earth, nanocrystal, fluorescence, in vivo bioimaging

## Abstract

In this study, Eu^3+^/Gd^3+^ co-doped fluoroapatitååe (Eu/Gd:FAP) nanocrystals were synthesized by the hydrothermal method as a fluorescent bioimaging agent. The phase composition, morphology, fluorescence, and biosafety of the resulting samples were characterized. Moreover, the in vivo fluorescent bioimaging application of Eu/Gd:FAP nanocrystals was evaluated in mice with subcutaneously transplanted tumors. The results showed that the Eu/Gd:FAP nanocrystals were short rod-like particles with a size of 59.27 ± 13.34 nm × 18.69 ± 3.32 nm. With an increasing F substitution content, the Eu/Gd:FAP nanocrystals displayed a decreased size and enhanced fluorescence emission. Eu/Gd:FAP nanocrystals did not show hemolysis and cytotoxicity, indicating good biocompatibility. In vivo fluorescent bioimaging study demonstrated that Eu/Gd:FAP nanocrystals could be used as a bioimaging agent and displayed stable fluorescence emitting in tumors, indicating an accumulation in tumor tissue due to the passive targeting ability. In addition, any adverse effects of Eu/Gd:FAP nanocrystals on major organs were not observed. This study shows that biocompatible rare earth co-doped FAP nanocrystals have the potential to be used as a bioimaging agent in vivo.

## 1. Introduction

Apatite is a family of minerals represented by calcium phosphate. The calcium phosphate apatite is the main inorganic component of the bones and teeth of mammals, which mainly exists as hydroxyapatite (HAP) in vivo [1,2]. With varied monovalent anions such as F^−^,Cl^−^, HAP can be changed to fluoroapatite (FAP) and chloroapatite (ClAP) [3,4,5]. Calcium phosphate apatite materials have been widely used in biomedical fields, such as bone repair and as drug/protein/gene carriers [6,7,8]. They have good biocompatibility, biodegradability, bioactivity, non-toxicity, non-immunogenicity, and osteoconductivity [9,10,11,12,13,14,15]. Furthermore, their properties can be adjusted depending on the ion composition. For example, FAP has a lower solubility and better acid resistance compared with HAP, contributing to protecting teeth from acid degradation [16,17,18,19,20].

In addition, the apatite lattice is very suitable for the doping of rare earth elements and can provide a suitable crystal field environment for promoting the fluorescence emission of rare earth ions located at the divalent cation sites of apatite [21,22,23,24,25]. So, the doping of rare earth elements can endow apatite unique fluorescent function. Based on narrow emission bandwidths, long fluorescence lifetime, and the excellent quantum yield of rare earth elements and the good biocompatibility and biodegradability of calcium phosphate apatite, rare-earth-doped apatite as a bioimaging agent has attracted attention [26,27]. Especially, FAP displays better host properties for rare earth ions than HAP. In apatite, F^−^ ions with lower vibration energy can significantly avoid the quenching effect of the -OH group to promote the fluorescence conversion of rare earth elements [28,29,30,31].

The high fluorescence of rare-earth-doped FAP is the first prerequisite for its use as a bioimaging agent. This can be improved by promoting the thermal diffusion of rare earth ions into the crystallographic site of Ca^2+^. In addition, utilizing the energy transfer between the co-doped rare earth elements is an alternative way to enhance the fluorescence [22,29]. For example, the luminescence emission could be sensitized by the successive energy transfer process from Gd^3+^ (excited state) to Eu^3+^ (ground state). Eu^3+^ ions are activated to the energy level that is matched with the ^6^G_J_ energy level of Gd^3+^. The Eu^3+^ ions could generate gradual energy level transitions to the ^5^H_J_, ^5^D_0_, and ^7^F_J_ of Eu^3+^ by radiation transition with low energy loss. Therefore, rare earth element co-doping is considered to be an effective method of improving the luminescence-emission intensity based on energy transfer and electron transfer processes [15]. Of course, the particle size of rare-earth-doped FAP also requires attention because particles that are too large are not conducive to cell uptake and further biological imaging [32]. The hydrothermal method can provide both high temperature and pressure in a sealed pressure vessel to promote the chemical reaction and form highly crystalline fluorapatite, enhancing the diffusions of co-doped rare earth elements into the lattice site, and simultaneously maintaining the fluorapatite crystals at the nanometer scale [3,33,34].

Herein, the Eu^3+^/Gd^3+^ co-doped fluoroapatite (Eu/Gd:FAP) nanocrystals were synthesized by the hydrothermal method based on the effect of energy transfer from Gd^3+^ to Eu^3+^. The phase composition, morphology, fluorescence, hemolysis, and cytotoxicity of the resulting samples were characterized by the varied fluoride ion (F^−^) proportions of apatite. The in vivo fluorescent bioimaging application of Eu/Gd:FAP nanocrystals was further carried out in mice with subcutaneously transplanted tumors, with the aim of revealing their potential in the bioimaging field.

## 2. Experimental Section

### 2.1. Materials

Calcium chloride dihydrate (CaCl_2_·2H_2_O, >99.9%), disodium hydrogen phosphate dodecahydrate (Na_2_HPO_4_·12H_2_O, >99.9%), europium nitrate hexahydrate (Eu(NO_3_)_3_·6H_2_O, >99.9%), gadolinium chloride hexahydrate (Gd(NO_3_)_3_·6H_2_O, >99.9%), and sodium fluoride (NaF, >99.9%) were purchased from Sinopharm Chemical Reagent Co., Ltd. Ultrapure water (>18 MΩ*cm, 25 °C) was used in the experiments. Human hepatocellular carcinomas (HepG2) and human normal hepatocyte cells (L02) were purchased from Qingqi (Shanghai) Biotechnology Development Co., Ltd. The BALB/c-nu mice and the rabbit were purchased from the Animal Experiment Center of Wuhan University.

### 2.2. Preparation of Eu^3+^/Gd^3+^ Co-Doped FAP Nanocrystals

The Eu^3+^/Gd^3+^ co-doped FAP (Eu/Gd:FAP) nanocrystals were synthesized by the hydrothermal method. The initial compounds were separately dispersed in deionized water (CaCl_2_·2H_2_O, Eu(NO_3_)_3_·6H_2_O, Gd(NO_3_)_3_·6H_2_O, Na_2_HPO_4_·12H_2_O, NaF). First, Na_2_HPO_4_·12H_2_O and NaF were dissolved together in water to achieve the mixing solution A of PO_4_^3−^ (0.24 mol/L) and F^−^ (0.08 mol/L). CaCl_2_·2H_2_O, Eu(NO_3_)_3_·6H_2_O, and Gd(NO_3_)_3_·6H_2_O were dissolved together in water to achieve the mixing solution B of Ca^2+^ (0.386 mol/L), Eu^3+^ (0.008 mol/L), and Gd^3+^ (0.006 mol/L). Then, solution A (15 mL) was poured into solution B (15 mL) and strongly stirred, and the NH_3_·H_2_O solution was added to the above suspension with a resulting pH of about 9–9.5. Thereupon, the suspension was transferred into a stainless-steel autoclave and treated at different temperatures (121 °C, 200 °C, and 300 °C) for 60 min. After cooling to room temperature, the resulting product was separated via centrifugation under 2000 rpm and washed with ultra-pure water three times. Finally, the product was dried using a freeze-drying method. In addition, the molar ratios of (Ca + Eu + Gd)/P, Eu/(Ca + Eu + Gd), and Gd/(Ca + Eu + Gd) were 1.67, 0.02, and 0.015, respectively.

### 2.3. Characterization of Samples

The morphology and elemental composition were investigated by using a scanning electron microscope (SEM, JEOL-7100F, Akishima, TKY, Japan) with energy dispersive spectroscopy (EDS) and a high-resolution transmission electron microscope (HRTEM, JEM-2100F, JEOL, Akishima, TKY, Japan). Crystalline phases and the phase composition were analyzed using a powder X-ray diffractometer (XRD, D/Max- IIIA, Rigaku Co., T Akishima, TKY, Japan). Fourier Transform Infrared (FT-IR, Thermo Nicolet 6700, Madison, WI, USA) Spectroscopy was used to identify the chemical structure and phase composition of the samples. The luminescence property was recorded with luminescence spectra using a 970CTR luminescence spectrophotometer (Shanghai Sanco, Shanghai, China). The equation calculates the relative enhancement ratio (*R*) of luminescence intensity:

$$R=\frac{I_{2}-I_{1}}{I_{1}}\times 100\%$$

where, *I*_1_ is the Eu/Gd:HAP emission peak intensity, and *I*_2_ is the Eu/Gd:FAP emission peak intensity.

### 2.4. Hemolysis and Cytotoxicity Evaluation

The hemolysis test was conducted using heparinized rabbit red blood cells (RBCs), and a 5% RBC suspension was prepared in a 0.9 wt% NaCl solution. The Eu/Gd:FAP suspension (1 mL) 0.9 wt% NaCl solution pre-incubated the RBC suspension. After that, the mixture was incubated at 37 °C for 1 h, and the supernatant was separated with centrifugation to check the light absorbance value (*OD*). Then, the RBCs were incubated with deionized water and 0.9 wt% NaCl for the control groups (positive and negative). The above results were used to calculate hemolysis % using the following equation:

$$Hemolysis\%=\frac{OD_{\mathrm{sample}}-OD_{\mathrm{neg}}}{OD_{\mathrm{pos}}-OD_{\mathrm{neg}}}\times 100\%$$

The cytotoxicity of the samples was observed using human normal hepatocyte cells (L02) and calculated using the following equation:

$$Inhibition\%=\frac{OD_{\mathrm{control}}-OD_{\exp}}{OD_{\mathrm{control}}}\times 100\%$$

where, *OD*_control_ is the absorbance value of the control group and *OD*_exp_ is the absorbance value of the experiment sample.

### 2.5. In Vivo Bioimaging

In vivo bioimaging was carried out using BALB/c-nu mice (about 20 g) with a subcutaneous transplantation of a HepG2 tumor. Eu/Gd/FAP powder was dispersed into ultrapure water as 1 mg/1 mL and injected into nude mice through a tail vein injection. An in vivo imaging test was conducted with a visible light imaging system (XRMSIII, Waltham, MA, USA) after 0.5 h, 1 h, 2 h, and 4 h. The mice in the control group were injected with the same volume of normal saline. Then, the tissues (heart, liver, spleen, pancreas, kidney, and lung) were taken out and washed three times with PBS solution. The procedure was continued for the tissue samples for imaging.

## 3. Results and Discussion

### 3.1. Characterization of Eu^3+^/Gd^3+^ Co-Doped Fluoroapatite

**Figure 1:**
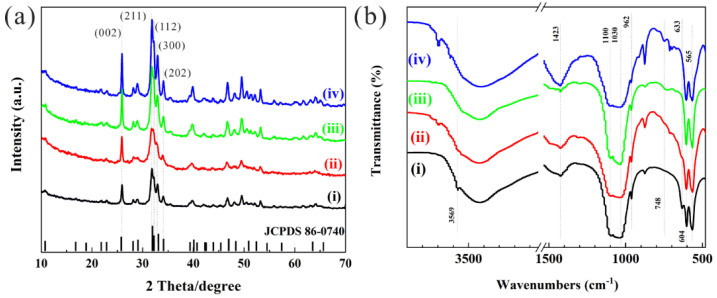
(**a**) XRD patterns and (**b**) FTIR spectra of Eu/Gd:FAP nanoparticles: (i) Eu/Gd:HAP; (ii) Eu/Gd:F(20%)AP; (iii) Eu/Gd:F(50%)AP; (iv) Eu/Gd:FAP.

The ratios of F substituted -OH are (i) 0%, (ii) 20%, (iii) 50%, and (iv) 100%, and for Eu/Gd substituted Ca is 2%/1.5%. The XRD results (Figure 1a) show that there is no obvious difference between the Eu/Gd:FAP and Eu/Gd:HAP nanoparticles. The characteristic diffraction peaks, (002), (211), (112), (300), and (202) are largely assigned to HAP (PDF JCPDS 86-0740) and FAP (PDF JCPDS 71-0880) with 2 theta degrees of 25.9, 31.9, 32.2, 32.9, and 34.1 degrees. With the increase in F substitution, the characteristic diffraction peaks, such as (211), (112), and (300), display some broadening or sharpening. For 20% F substitution, the broadening of the diffraction line could be due to crystallization inhibition by F. While, increased F substitution (50% and 100%) causes increasing diffraction intensity and sharper diffraction peaks, which infers a gradual change in the main crystalline phase from HAP to FAP with increasing crystallinity. It can be confirmed that small doping of the Eu/Gd element has little effect on the characteristic diffraction peaks, and FAP shows higher crystallinity than HAP. FT-IR spectra (Figure 1b) reveal the characteristic vibrational peaks of HAP at 3569 cm^−^^1^ and 631 cm^−^^1^ (-OH stretching vibration and bending vibration), 1100 cm^−^^1^ and 1030 cm^−^^1^ (ν_3_ PO_4_^3−^ asymmetric stretching), 603 cm^−^^1^, 564 cm^−^^1^ (ν_4_ PO_4_^3−^ antisymmetric stretching), and 962 cm^−^^1^ (ν_1_ PO_4_^3−^ asymmetric stretching). The ionic radius of the F^−^ ions are smaller than that of the -OH group, but its electronegativity is higher than the latter. The crystallinity increase (Figure 1a), and the vibration strength of -OH decreases, which indicates the increasing F substitution content. Moreover, a new bond vibration is detected at 748 cm^−1^ for this. The vibrational peaks of the PO_4_^3−^ group in the product can be identified at 1100 cm^−1^, 1030 cm^−1^, 962 cm^−1^, 564 cm^−1^, and 603 cm^−1^. Moreover, the peak at 876 cm^−1^ represents HPO_4_^2−^, which enhances with the substitution of hydroxyl fluoride, as well as a peak at 1423 cm^−1^, which indicates the presence of carbonate (vibrations of carbonate) and which means the sample was partially carbonized.

**Figure 2:**
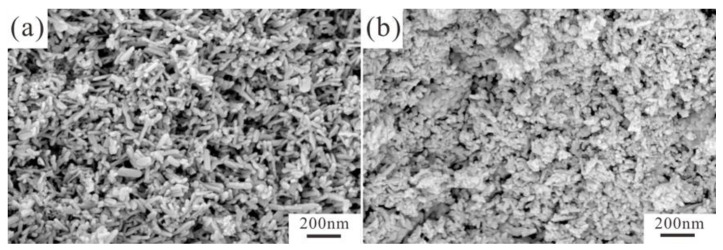
SEM images of (**a**) Eu/Gd(2/1.5):HAP and (**b**) Eu/Gd(2/1.5):FAP.

Figure 1 displays the XRD patterns and FTIR spectra of samples.The as-dried Eu^3+^/Gd^3+^ (2%/1.5%) co-doped HAP (Figure 2a) and FAP (Figure 2b) nanoparticles are shown in Figure 2. It has been suggested that F substitution leads to a reduction in the length of the rod-like nanoparticles, which is consistent with our previous report [31]. The average diameter × length size of Eu/Gd:FAP is 59.27 ± 13.34 nm × 18.69 ± 3.32 nm, obtained by calculation and statistics no less than fifty times. From the morphology of the samples, HAP (99.53 ± 21.03 nm × 24.29 ± 3.70 nm) is larger than FAP in crystal size, and it could be the smaller aspect ratio of the rod-shaped particles for FAP (3.17, 4.10 for HAP). In addition, the agglomeration of FAP is higher than HAP.

**Figure 3:**
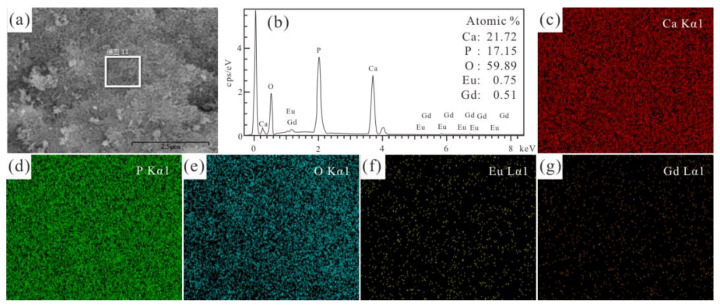
(**a**) SEM image, (**b**) EDS spectrum analysis results and its elements mapping: (**c**) Ca, (**d**) P, (**e**) O, (**f**) Eu and (**g**) Gd of the marked regions in (**a**) of Eu/Gd(2/1.5):HAP.

**Figure 4:**
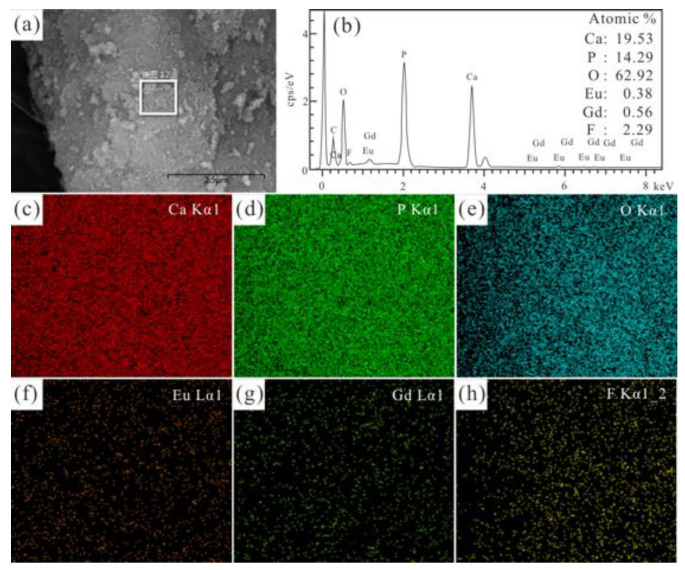
(**a**) SEM image, (**b**) EDS spectrum analysis results and its elements mapping: (**c**) Ca, (**d**) P, (**e**) O, (**f**) Eu, (**g**) Gd and (**h**) F of the marked regions in (**a**) of Eu/Gd(2/1.5):FAP.

As shown in Figure 3 and Figure 4, the EDS results of the selected area (in Figure 3a and Figure 4a) reveal the presence of Ca, P, O, Gd, Eu, and F in the Eu/Gd:HAP and Eu/Gd:FAP product. The molar ratios of (Ca + Eu + Gd)/P of Eu/Gd:HAP and Eu/Gd:FAP (Figure 3b and Figure 4b) are 1.34 and 1.43, respectively. In Figure 3c–g and Figure 4c–h, the EDS scan mapping of each element are stochastic and uniform. Furthermore, the EDS mapping of Eu and Gd (Figure 3g,h) unveils that those doping elements have successfully entered into the crystal lattice of the product, as well as the F substitution element.

**Figure 5:**
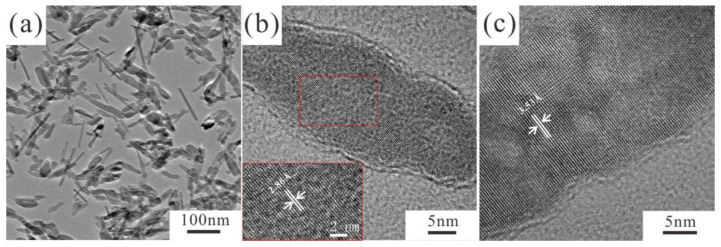
(**a**) TEM image and (**b**,**c**) HRTEM images of Eu/Gd:FAP. Inset in (**b**): magnified HRTEM image of a single crystal with a d-spacing of 2.86 Å.

TEM images of Eu/Gd co-doped FAP help to widen the size information as well as microstructures. The morphology of Eu/Gd:FAP shows rod-shaped nanoparticles. The fluoroapatite crystal structure is presented, and (201) and (211) lattice planes with an interplanar spacing of 3.53 Å and 2.86 Å are assigned in high-resolution TEM images (inset in Figure 5b,c).

### 3.2. Fluorescence Properties

**Figure 6:**
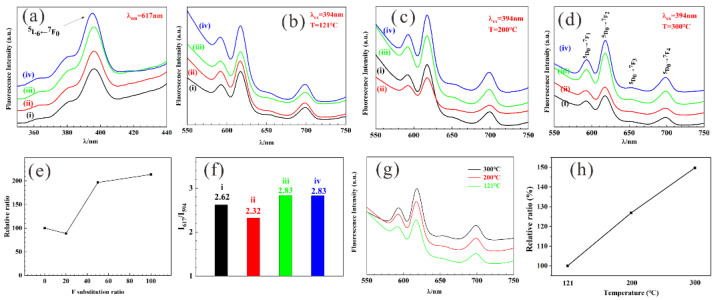
(**a**) Excitation spectra (λ_em_ = 617 nm) and fluorescent emission spectra (λ_ex_ = 394 nm) of product at (**b**) 121 °C, (**c**) 200 °C, (**d**) 300 °C. (**e**) Luminescence enhancement relative ratio and (**f**) I_591_/I_617_ value at 300 °C for different F substitution. (**g**) Fluorescent emission spectra and (**h**) luminescence enhancement relative ratio of Eu/Gd:FAP at different temperatures. (i) Eu/Gd:HAP; (ii) Eu/Gd:F(20%)AP; (iii) Eu/Gd:F(50%)AP; (iv) Eu/Gd:FAP.

The fluorescence properties of rare earth elements would be significantly promoted by doping into an apatite lattice, which could provide a hydrophobic environment from fluorescence quenching and a lowered crystal symmetry environment for higher fluorescence emission. The trivalent europium ion has been a common choice in past research for a lanthanide fluorescence probe with a special 4f^6^ electronic configuration and a strong red luminescence in the visible spectral region. The intensive excitation wavelength is centered at 394 nm in Figure 6a, contributing to the ^5^L_6_←^7^F_0_ energy level transition [35]. The strongest four emission peaks (in Figure 6b–d) are 591 nm, 617 nm, 651 nm, and 699 nm, assigned to ^5^D_0_→^7^F_1_, ^5^D_0_→^7^F_2_, ^5^D_0_→^7^F_3_, and ^5^D_0_→^7^F_4_ energy level transitions, respectively. Luminescence emission intensity would slightly decline (in Figure 6e) due to F ions (20% substitution) inhibiting HAP crystallization. When FAP is the main crystalline phase, that is, 50% and 100% F substitution, higher emissions will be recorded. This is a result consistent with the above XRD analysis.

The ^5^D_0_→^7^F_1_ transition, magnetic dipole transition at 591 nm, is an invariable integrated intensity less influenced by Eu^3+^ surroundings. While, the ^5^D_0_→^7^F_2_ transition is extremely dependent on the vicinity of Eu^3+^ ions, the so-called hypersensitive transition. In general, the symmetry/asymmetry for Eu^3+^ can be instructed by the ratio of I_617_/I_591_ [36], where I_591_ and I_617_ refer to the intensity of ^5^D_0_→^7^F_2_ and ^5^D_0_→^7^F_1_ transition, respectively. The I_617_/I_591_ value is listed in Figure 6f. For 20%, the F substitution is smaller than others, which could be due to lower crystallinity, as mentioned in Figure 1a. Moreover, it was observed that Eu/Gd:FAP, which are prepared at different hydrothermal temperatures (Figure 6g), can also obviously promote the fluorescence properties, as shown in Figure 6h. According to the results, the higher temperature (300 °C) is the better temperature for the material preparation due to the increasing crystallinity of the products relating to the thermal diffusion of Eu^3+^ to the Ca^2+^ sites in the crystal lattice [3].

### 3.3. Biosafety Evaluation

**Figure 7:**
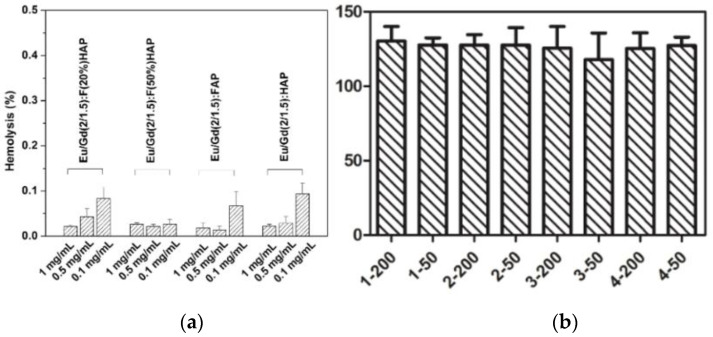
(**a**) Hemolysis percentage of red blood cells. (**b**) Cytotoxicity of L02 cells incubated with various concentrations samples for 3 h. Sample 1: Eu/Gd:F(20%)HAP, Sample 2: Eu/Gd:F(50%)HAP, Sample 3: Eu/Gd:FAP, Sample 4: Eu/Gd:HAP; 200 and 50 means the concentrations co-cultured with cells, unit is μg/mL.

As shown in Figure 7, it was carried out by hemolysis and cytotoxicity tests. No distinct hemolysis is observed, and the hemolysis rate is less than 0.1% (Figure 7a) for concentrations as high as 1 mg/mL during 3 h. The cell viability of the samples is far greater than 100%, suggesting non-cytotoxicity to L02 cells (co-cultured concentrations up to 200 μg/mL). The results both show the advantages of biological safety for apatite, so the material tends to be used as a bioimaging agent in the biomedical field.

### 3.4. In Vivo Imaging

**Figure 8:**
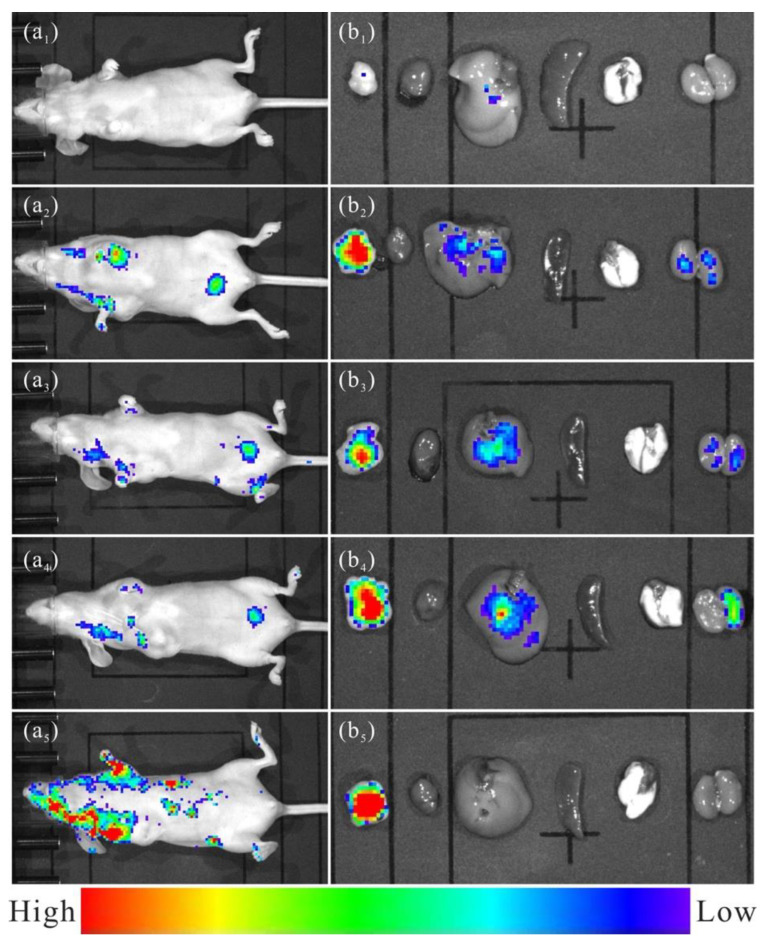
Fluorescent imaging of (**a**) nude mice with subcutaneous transplantation tumor (HepG2) after injecting Eu/Gd:FAP samples and (**b**) Extracted organs after euthanasia (from left to right): tumor, heart, liver, spleen, lung, and kidney. (**a_1_**,**b_1_**) control group, (**a_2_**,**b_2_**) 0.5 h, (**a_3_**,**b_3_**) 1 h, (**a_4_**,**b_4_**) 2 h and (**a_5_**,**b_5_**) 4 h.

**Figure 9:**
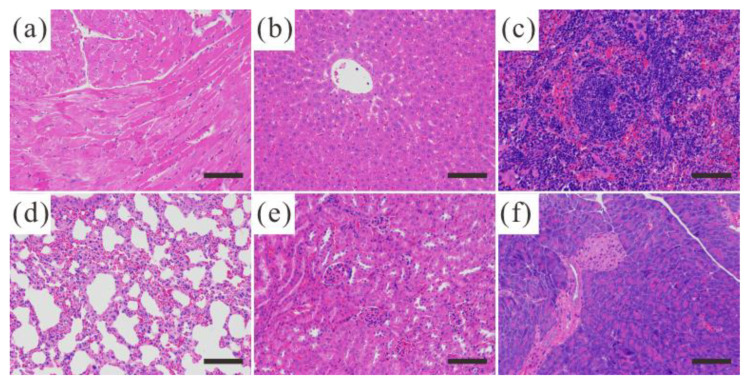
Hematoxylin and eosin (H&E) staining tissue sections of different organs: (**a**) heart, (**b**) liver, (**c**) spleen, (**d**) lung, (**e**) kidney, and (**f**) pancreas. Scale bar is 100 μm.

In vivo fluorescent imaging was conducted, as shown in Figure 8. Compared with the blank control group, the Eu/Gd:FAP treated groups have a significant fluorescent imaging signal, which means that the material will enter the organs in vivo and act as a fluorescent bioimaging agent. There was no significant signal in these tissues in the heart, spleen, and lung, but a weak signal in the liver and kidney, as seen in Figure 8b. An intense radiance signal was detected after the tail was intravenously administered for 0.5 h. Moreover, the fluorescence signal lasts for a long time and has no remarkable attenuation within 4 h. Moreover, the weak signal in the liver, kidney, and spleen infers a higher clearance by the reticuloendothelial system (RES) [37,38]. A nonremarkable accumulation in healthy tissues/organs, quick uptake, or scavenging by RES are the potential advantages of nano-biomaterials in clinical settings. Furthermore, this could be confirmed by gradually increasing the radiance signal on the mouse’s neck and armpit, areas with abundant lymphatic systems (Figure 8a). Importantly, the tumor tissue of the Eu/Gd:FAP-treated groups, with their stronger and longer radiance signals, indicate higher accumulation owing to the enhanced permeation and retention effect [39,40]; thus, it also may be used as a tumor-targeting material for delivering anti-cancer drugs for an integrated diagnosis and treatment platform. The organs and tissues of the nude mice display no apparent abnormality and damage by observing the section (hematoxylin and eosin staining, in Figure 9).

## 4. Conclusions

In summary, we successfully prepared Eu/Gd co-doped fluoroapatite nanocrystals as a luminescent-enhancing agent. Due to F substitution, the resulting Eu/Ga:FAP has a smaller aspect ratio, higher crystallinity, and luminescence emission, suggesting the transformation of the main crystalline phase from HAP to FAP. With increasing hydrothermal temperatures, the crystallinity of Eu/Ga:FAP is further improved. Moreover, the fluorescence properties are significantly enhanced as a result of Eu^3+^ asymmetrical surroundings. The biosafety was verified by conducting hemolysis and cytotoxicity tests, and the tissue sections also showed no damage or abnormality. In comparison to healthy organs, Eu/Gd:FAP has a much higher accumulation in tumors, with a stronger and longer radiance signal. These findings contribute in several ways to our understanding of rare earth doped FAP and provide the basis for further research. More research is required to examine the long-term efficacy and safety of the material.

## Data Availability

Data are available upon reasonable request.
